# Unilateral and bilateral ex vivo cleft lip models: universal tools for surgical training at different levels of experience

**DOI:** 10.1186/s13005-025-00565-y

**Published:** 2025-12-04

**Authors:** Christoph Vogl, Katja L. Schulz, Manuel Olmos, Manuel Weber, Tobias Möst, Marco R. Kesting, Rainer Lutz

**Affiliations:** 1 Department of Oral and Cranio-Maxillofacial Surgery, Friedrich-Alexander-Universität Erlangen-Nürnberg (FAU), University Hospital Erlangen, Erlangen, Germany; 2 Philipps-Universität Marburg, Marburg, Germany; 3 Department of Oral and Cranio-Maxillofacial Surgery, UKGM GmbH, University Hospital Marburg, Baldingerstr., Marburg, 35043 Germany

**Keywords:** Unilateral cleft lip, Bilateral cleft lip, Porcine snout disc, Ex vivo model, Surgical training, Ex vivo cleft lip model, Cleft lip surgery

## Abstract

**Introduction:**

Our group has recently established the first ex vivo cadaveric models to simulate unilateral and bilateral cleft lip surgery. We have shown that these porcine snout disc models are suitable for novice cleft surgeons to understand the basic principles of cleft lip surgery and to practice the essential surgical steps. In this study we want to show that the ex vivo cleft lip models are not only suitable and helpful for residents, but also for students and experienced surgeons with and without experience in cleft surgery.

**Methods:**

Three courses were held to evaluate the ex vivo cleft lip models: One for oral and maxillofacial surgeons from German cleft centers and two courses for dental students. All operated on the unilateral and bilateral ex vivo cleft lip models using the Millard technique. Questionnaires were used to assess their subjective opinion of the ex vivo cleft lip models. Tasks for the students were carried out to objectify their learning success. Three-dimensional scans were taken and analyzed using a previously validated intraoral scanner to objectively assess the outcome.

**Results:**

The ex vivo cleft lip models are suitable for the training of students and experienced surgeons with varying levels of experience in cleft lip repair. Both models were rated as realistic in terms of tissue resemblance, surgical planning, and surgical management. In addition, the specialists rated the models as realistic overall. The models also provide students with several advantages, such as improved surgical skills and a better understanding of cleft surgery. Participants felt that the models should be made available not only to residents, but also to consultants and senior consultants and should be implicated in the dental curriculum.

**Conclusion:**

The ex vivo cleft lip models made of porcine snout discs are suitable for a wide range of users starting from students to experienced surgeons to train in cleft lip surgery. The great advantage of these models is that experienced surgeons can work with an inexpensive and readily available model to improve their skills and modify their techniques.

## Introduction

 Cleft lip surgery is a demanding procedure in a highly aesthetic region with lifelong impact for patients. It requires experienced surgeons, yet training opportunities remain limited. Surgical models can help address this gap, but their cost and fidelity vary considerably [1]. High-fidelity models, unlike low-fidelity ones, reproduce the multilayered anatomy of skin and muscle, which is critical for accurately reconstructing cleft structures [2]. However, existing silicone models are scarce and often prohibitively expensive [1]. To overcome this limitation, our group developed ex vivo unilateral and bilateral cleft lip models using porcine snout discs [3, 4]. Naturally multilayered, they provide realistic haptic feedback at low cost [3, 4]. In previous work, we demonstrated their use among residents in oral and maxillofacial surgery, who successfully performed key steps such as orbicularis oris reattachment, vermillion repair, and C-flap preparation [3, 4]. Residents reported significant gains in both theory and practice and advocated wider availability of the models [3, 4]. We concluded that these ex vivo models are suitable for novice surgeons, aligning with the teaching mission of academic hospitals [3, 4]. 

Building on this, we presented the models to experienced specialists to assess their realism, strengths, and limitations, and to test their application at higher levels of surgical expertise. We also evaluated their suitability as teaching aids for dental students with limited exposure. Because early and repeated integration of theory and practice enhances surgical learning, porcine snout models offer a safe, inexpensive, and repeatable environment for trainees. They allow mistakes, corrections, confidence-building, and skill optimization without patient risk—often a more effective path to mastery than error avoidance alone [5–7]. 

Beyond skill acquisition, accurate outcome evaluation is essential in cleft surgery. While photographs have long been used for documentation, three-dimensional analysis offers superior accuracy and is increasingly emphasized [8, 9]. For clinical feasibility, intraoral scanners are particularly suitable for imaging the nasolabial region of cleft models, as we have recently shown. Simulation on high-fidelity models, combined with 3D analysis, also enables objective assessment of surgeon competence [10, 11]. We therefore included 3D evaluation of surgical outcomes on the unilateral and bilateral models performed by specialists.

This study aims to determine whether ex vivo cleft lip models improve both theoretical understanding and practical skills in cleft surgery—not only for residents, but also for specialists and dental students.

## Methods

The detailed development and initial evaluation of the ex vivo porcine snout disc models for unilateral and bilateral cleft lip surgery have previously been described by Lutz et al. [3, 4] All porcine snout discs were obtained from a local slaughterhouse. Each participant received two snout discs (one for unilateral and one for bilateral cleft lip closure). To compensate for potential damage to some snouts during the slaughter process, additional specimens were acquired for each course to ensure sufficient quality models were available.

## Course program

### Specialists in oral and cranio-maxillofacial surgery

A workshop for specialists in oral and maxillofacial surgery was held in Erlangen, Germany, in March 2023 on the topic of “Cleft Lip Surgery.” During this meeting, the ex vivo cleft lip models described by Lutz et al. were introduced, along with step-by-step demonstrations of the Millard technique for both unilateral and bilateral cleft lip repair. To better understand which landmarks on the porcine snout correspond to the landmarks on the human nasolabial region, the participants created the unilateral model with all the important landmarks themselves. Under the supervision of two senior cleft surgeons, ten participants performed the Millard technique on porcine snout discs step by step.

### Dental students

Two courses for dental students were conducted, in April 2024 and February 2025. Both courses consisted of a theoretical introduction to cleft surgery followed by hands-on training with the ex vivo models. In the 2024 course, the theoretical instruction and practical exercise were delivered online. Eleven students simulated cleft closure at home using the porcine models and disposable paper templates. In the 2025 course, 17 students participated on-site at our clinic. The course included the same theoretical instruction as the first iteration, followed by guided practical exercises. Three teaching assistants were available to answer questions during the hands-on session. In both courses, cleft lip closure was taught using step-by-step instructions. At the time of participation, students were in their fourth year of dental studies and had completed all required surgical coursework. Each student attended only one course.

## Subjective evaluation

### Questionnaires for specialists

Before and after performing the surgical simulation, specialists were asked to complete a self-assessment questionnaire. A numerical scale ranging from 0 (“does not apply”) to 10 (“applies completely”) was used. The questionnaire assessed previous experience in cleft surgery and self-perceived competence. Separate questionnaires were used for the unilateral and bilateral models; for clarity, Table 1 provides a summary combining the items from both versions into one unified list. The original questionnaires were administered in German and later translated into English for this paper.

**Table 1:** Specialist questionnaire: questions asked separately for unilateral and bilateral ex vivo cleft lip models

Questions on the ex vivo cleft lip models for specialists
Possible answers from 0–10: “0 = does not apply”, “5 = partly”, “10 = applies completely”
I already have general theoretical previous knowledge of cleft surgery
I know the procedure for unilateral/bilateral cleft lip closure in theory
I already have practical experience in unilateral/bilateral cleft lip surgery
My general understanding of cleft lip surgery was improved by practicing on the model
My theoretical general knowledge of unilateral/bilateral cleft lip surgery was improved by the practice model
Only for specialists and senior physicians with prior knowledge in cleft surgery: I was able to deepen/consolidate my previous knowledge through practical exercise
My overall practical skills regarding unilateral/bilateral cleft lip surgery were improved by the practice model
I consider the model overall a suitable exercise to improve my skills in cleft surgery
The model should be made available to every student, resident, and specialist and senior physician
Only for specialists and senior physicians with previous experience with other surgical models for cleft surgery: this model has a clear added value compared to previous models
Possible answers from 0–10: “0 = very poor”, “5 = pass”, “10 = excellent”
Tissue resemblance: How well could the porcine model simulate the anatomical and tissue properties of the human nasolabial region
Realistic planning and surgical procedure: How well could the porcine model simulate preoperative planning and the surgical procedure Overall realism: General realism of the model

To evaluate the event, another questionnaire was sent to the participants with the questions listed in Table 2

**Table 2:** Questions about the event itself

Questions on the event
Possible answers from “1 = not helpful” to “5 = very helpful”
The case studies presented were…
The presentations were…
Overall, the theoretical part was…
The presentation on the unilateral cleft model was…
The presentation on the bilateral cleft model was…
Possible answers “1 = too short”, “2 = short”, “3 = appropriate”, “4 = long”, “5 = too long”
The temporal scope of the case studies was…
The temporal scope of the lectures was…
Overall, the temporal scope of the theoretical part was…
The time to work with the unilateral cleft model was…
The time to work with the bilateral cleft model was…
Overall, the temporal scope of the practical part was…

### Questionnaires for dental students

As for the specialists, the students received a questionnaire adapted to their knowledge (Table 3).

**Table 3:** Questionnaire for students: questions asked separately for unilateral and bilateral ex vivo cleft lip models

Questions on the ex vivo cleft lip models for students
Possible answers from 0–10: “0 = does not apply”, “5 = partly”, “10 = applies completely”
I already have general theoretical previous knowledge of cleft surgery
I know the procedure for unilateral/bilateral cleft lip closure in theory
I already have practical experience in unilateral/bilateral cleft surgery
My general understanding of cleft surgery was improved by practicing on the model
My theoretical general knowledge of unilateral/bilateral cleft lip surgery was improved by the practice model
I was able to deepen/consolidate my previous knowledge through the practical exercise
My overall practical skills regarding unilateral/bilateral cleft lip surgery were improved by the practice model
I consider the model overall a suitable exercise to improve my skills in cleft surgery
The model should be made available to every (dental) student
I would generally like to see more surgical exercises on cleft surgery in my studies

## Objective assessment

**Fig. 1:**
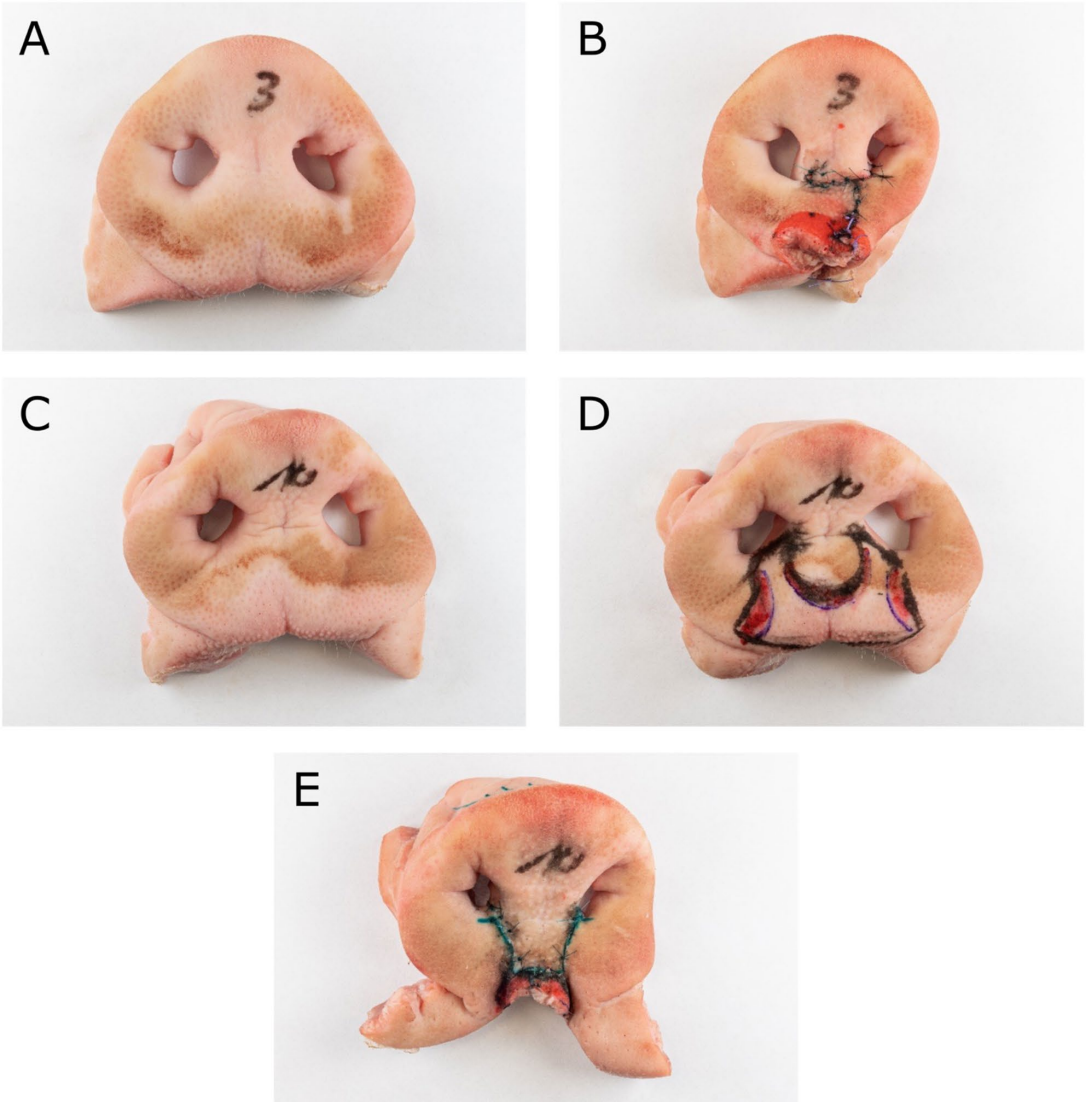
Photograph of the porcine snout disc before surgery (**A**) and after completion of the Millard II procedure (**B**) in the unilateral ex vivo cleft lip model. Figures 1C-D show the bilateral ex vivo cleft lip model before surgery (**C**), after marking (**D**) and after the Millard procedure (**E**)

For the objective assessment photographs (Fig. 1) and 3-dimensional scans (Trios 4, 3Shape, Copenhagen, Denmark) of the pigs’ snouts were taken before and after the simulations in all courses.

### Three-dimensional measurements for specialists

In order to objectively evaluate the success of the unilateral cleft lip surgery simulations, the postoperative lengths of the philtrum edges were measured and compared with the preoperative situation in the specialist course. Specialists have more experience, so a closer look at the results using 3-dimensional scans seems useful to make a precise analysis of the level of experience in cleft surgery.

**Fig. 2:**
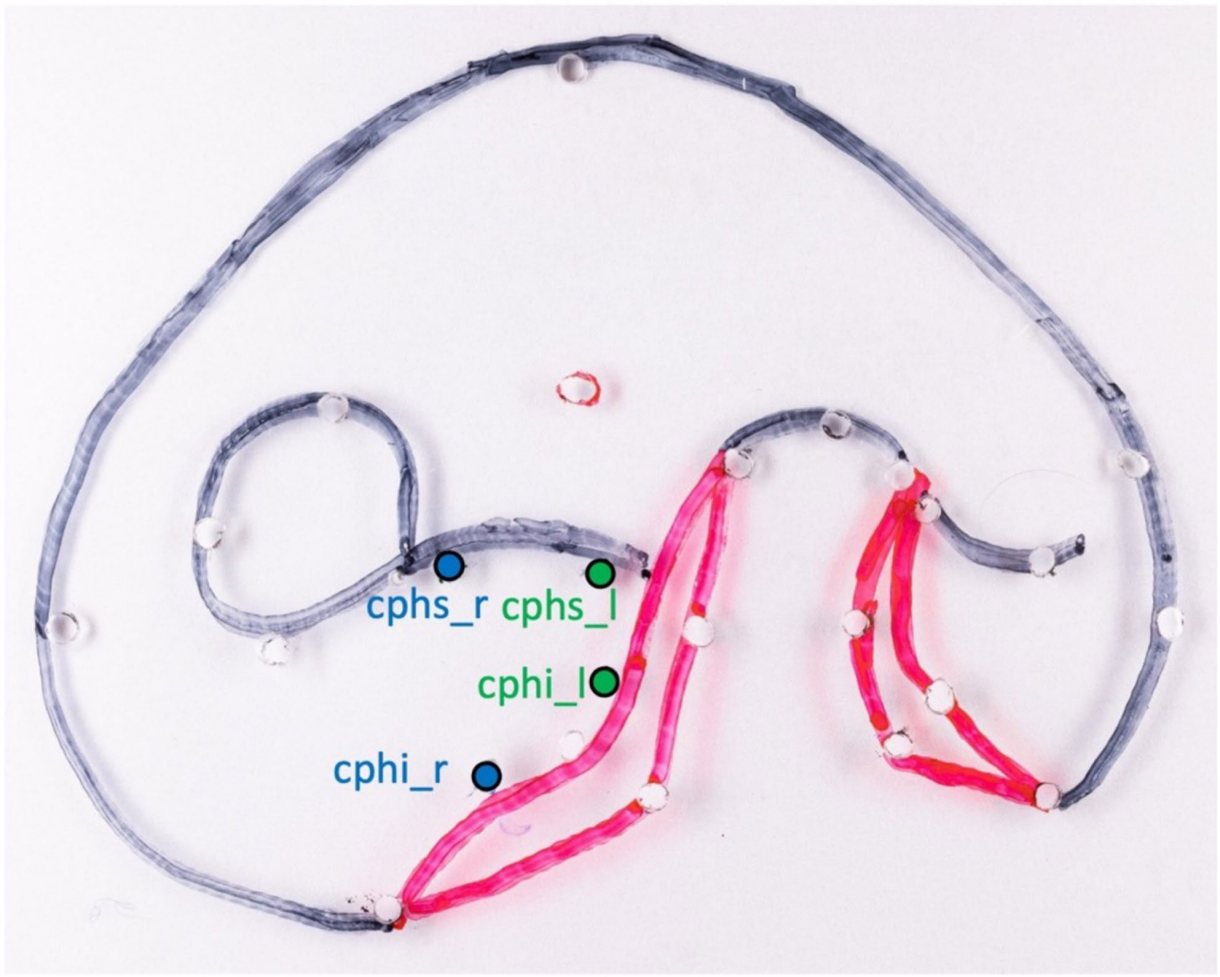
Foil template of the unilateral cleft model. The points of the right (blue) and left (green) philtrum columns are marked. The crista philtri superior (*cphs*) is defined as the highest point of the philtrum column and the crista philtri inferior (*cphi*) as the peak of the cupid bow

**Fig. 3:**
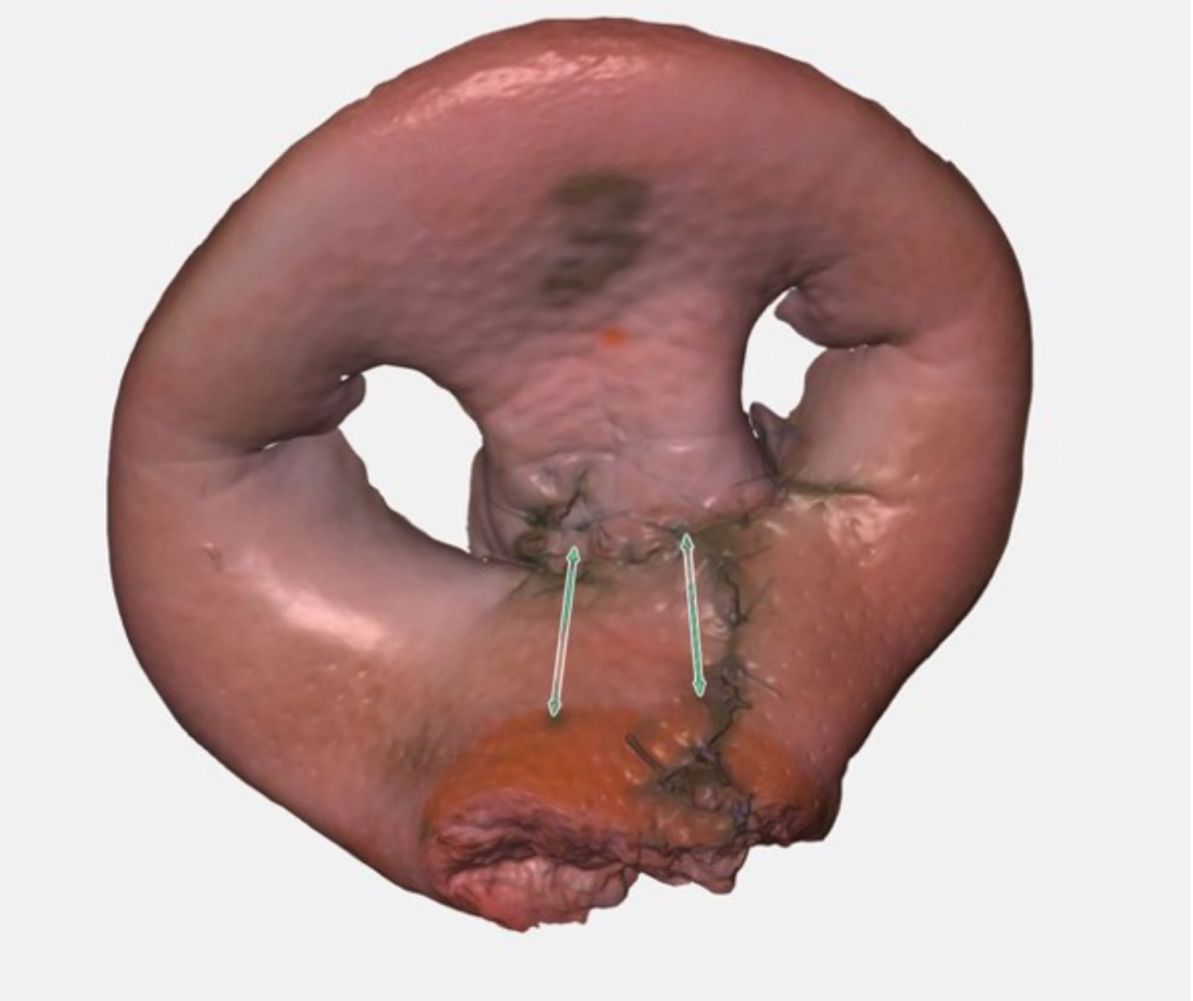
3-dimensional scan of the ex vivo cleft lip model after unilateral cleft lip repair (Millard II). The arrows indicate to the points cphi and cphs, which are used to measure the lengths of the right and left philtrum edges

The distances of points cphi_r and cphs_r on the non-cleft side and cphi_l and cphs_l on the cleft side of the template were taken as the preoperative baseline for the philtrum lengths (Fig. 2). The reconstructed philtrum edges were measured on the postoperative 3D scans.

(.ply format) as both direct and volumetric distances. The 3D evaluation and measurement tool Zeiss Inspect (Co. Zeiss Inspect, Oberkochen, Germany) was used for this purpose (Fig. 3). The measured postoperative distances were then compared with the preoperative baseline. An important goal of cleft lip surgery is to achieve symmetry of the philtrum. Therefore, the differences in philtrum length between cleft and non-cleft were calculated and the results achieved by the participants were assessed to objectify the surgical outcome. All measurements were reviewed by an experienced cleft surgeon and modified as needed to ensure precision.

In addition, the participants’ scores were divided into two groups to assess whether different skill levels could be distinguished when assessing the surgical outcomes: participants who reported little previous experience with unilateral cleft surgery (score on questionnaire < 5) were differentiated from more experienced surgeons (score on questionnaire $\:\ge\:$5). More experienced surgeons were expected to achieve better symmetry of the philtrum margins, represented by a smaller difference in the length of the non-cleft and cleft philtrum margins.

### Rating of the students´ surgical outcomes

Since the students have little surgical experience and knowledge of cleft surgery, their simulation results were evaluated only on the basis of the photographs. Therefore, two observers, who were familiar with the models, rated the final results of the surgical simulation The results were subsequently checked by an experienced cleft surgeon. The scale had three levels:

- 0 = the participant couldn´t finish the cleft closure or the lip plasty was anatomical incorrect.
- 1 = the provided model has minor anatomical mistakes.
- 2 = the provided model is anatomical correct, the model is finished or nearly finished.

The average rating of both observers was used for further analysis.

### Knowledge tests for students

**Fig. 4:**
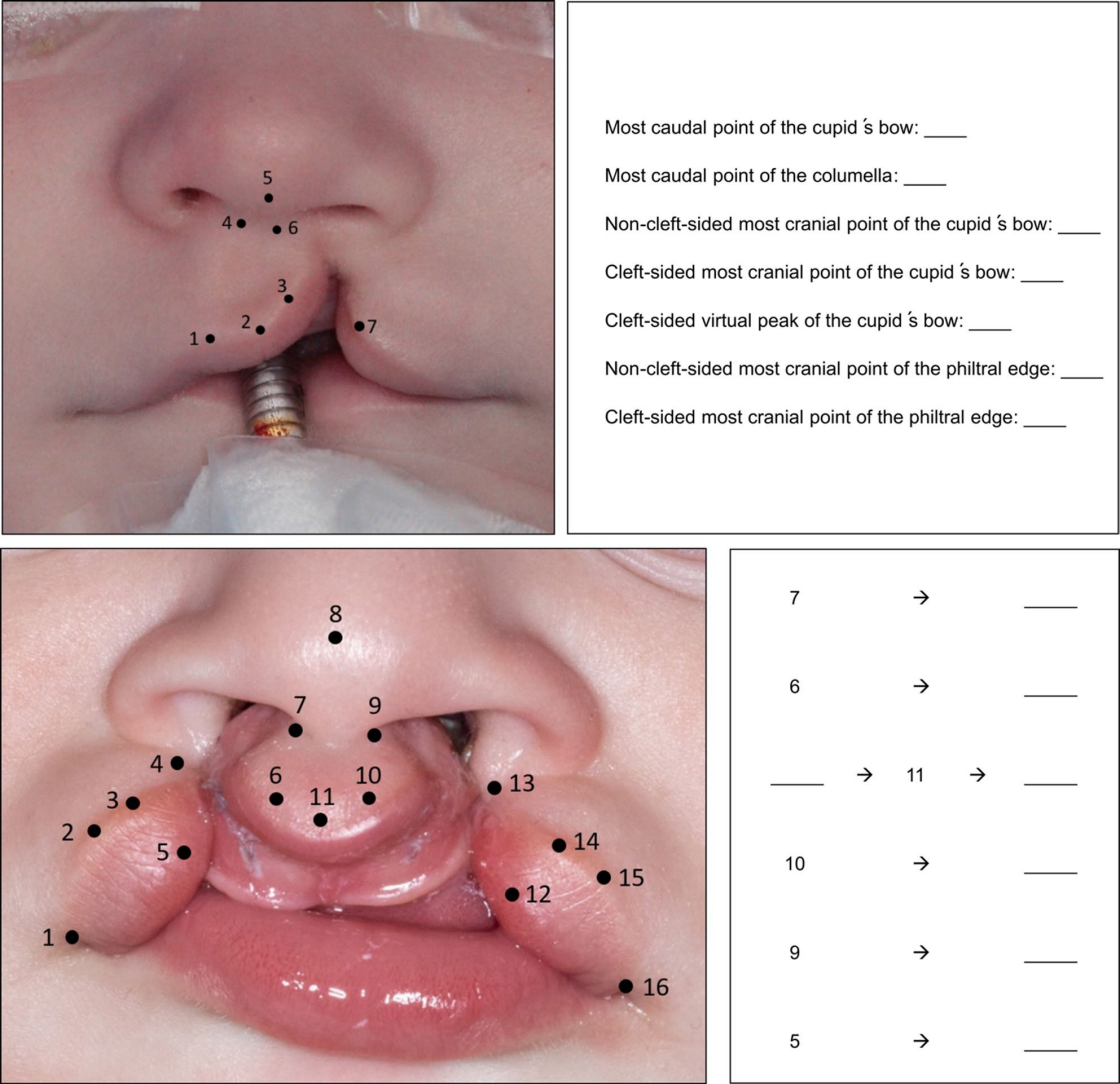
In the first task (**A**) the students had to match the anatomical landmarks with the numbers in the picture on the left. Another task (**B**) was to match the numbered landmarks in the way they would merge during closure of a bilateral cleft lip

To be able to objectively assess the learning success, the students were given two tasks to complete before the start and after the end of the face-to-face course day (Fig. 4). Both the questionnaires and the tasks were in German and were translated for the paper.

### Statistical analysis

To analyze the 3-dimensional measurements the Shapiro-Wilk test was used to test for normal distribution. Independent samples t-test was used to evaluate the differences between the two groups. The p-value was set at 0.05 for all analyses. SPSS 28 (IBM Corp., Armonk, New York, USA) was used for statistical analysis. All calculations were carried out by two independent observers. The mean value and the standard deviation were calculated for the evaluation of the questionnaires and the surgical outcome of the cleft closure performed by the students. For analyzing the knowledge tests the mean value was calculated.

## Results

Participants in this study generally rated their theoretical knowledge of cleft surgery as high (average 8.3, SD 2.00, *n* = 10).

## Specialists course

### Evaluation of the unilateral ex vivo cleft lip model

The specialist evaluation of the unilateral cleft lip model is shown in Table 4. Prior to the surgical simulation, the participants reported that they had generally mastered the theoretical principles of unilateral cleft lip closure (average 8.0, SD 2.49). In contrast, practical experience with unilateral cleft lip closure was lower (average 5.30, SD 3.77). The porcine snout model improved the participants’ self-assessed overall understanding of cleft surgery (average 9.50, SD.71). Not only theoretical knowledge (average 7.5, SD 2.76) but also practical skills (average 8.3, SD 3.06) were reported to be improved by the training. According to the participants, the model is suitable for improving their skills in cleft surgery (average 8.4, SD 3.06) and offers clear advantages over other models of cleft surgery (average 9.33, SD 1.16). One participant commented on the questionnaire that he was not aware of cleft surgery models before the course. The survey showed that participants felt that the ex vivo cleft lip models should be accessible to a wide range of people, including students, residents, specialists, and senior consultants (average 9.7, SD 0.66). The assessment of the similarity of the porcine snout model to the anatomical structures and tissue characteristics of the human nasolabial region showed good results (average 7.00, SD 1.01). The model was also rated as sufficient for a realistic surgical planning and execution (average 7.80, SD 1.23). It was rated similarly for its overall realism (average 7.70, SD 1.25).

The linear measurements of the philtrum edges are shown in Table 4 for all participants and for the two subgroups. We measured not only the direct distance between the points listed above, but also the corresponding distance on the surface between these points (Table 5).

**Table 4:** Length of the philtrum edges before and after surgery and the changes achieved for all participants and for the two subgroups

Length of philtrum edge (direct distance) in mm		On the cleft side	On the non-cleft side	Difference between cleft and non-cleft side
Absolute values before surgery		7.65	15.27	−7.62
Absolute values after surgery	All participants (*n* = 10)	11.28 ± 1.40	14.62 ± 1.67	−3.33 ± 2.25
	Inexperienced surgeons (*n* = 6)	11.26 ± 1.55	14.67 ± 2.09	−3.42 ± 2.77
	Experienced surgeons (*n* = 4)	11.33 ± 1.38	14.53 ± 1.02	−3.21 ± 1.52
Changes achieved	All participants (*n* = 10)	+ 3.63 (+ 47%)	−0.65 (−4%)	−4.29 (−56%)
	Inexperienced surgeons (*n* = 6)	+ 3.61 (+ 47%)	−0.60 (−4%)	−4.20 (−55%)
	Experienced surgeons (*n* = 4)	+ 3.68 (+ 48%)	−0.74 (−5%)	−4.41 (−58%)

**Table 5:** Volumetric distances of the philtrum edge on the cleft and non-cleft side after surgery, divided into inexperienced, experienced and all participants

Length of philtrum edge after surgery (volumetric curves), absolute values in mm	On the cleft side	On the non-cleft side		Difference between cleft and non-cleft side
All participants (*n* = 10)	11.86 ± 1.33	15.43 ± 1.61	−3.57 ± 2.09	
Inexperienced surgeons (*n* = 6)	11.86 ± 1.42	15.62 ± 2.01	−3.77 ± 2.49	
Experienced surgeons (*n* = 4)	11.87 ± 1.39	15.15 ± 0.91	−3.29 ± 1.60	

All measured distances were normally distributed (*p* > 0.05). Statistical analysis showed no significant differences in the measured outcomes for the two subgroups with different self-reported skill levels (*p* > 0.05).

### Evaluation of the bilateral ex vivo cleft lip model

Eight specialists in oral and maxillofacial surgery took part in the bilateral cleft lip surgery. The mean responses to the questionnaire are shown in Table 4. They reported an average theoretical experience of 8.12 (SD 2.85) and an average practical experience of 4.00 (SD 5.01) in bilateral cleft surgery. The model improved their knowledge (average 8.00, SD 3.61) and their perceived understanding of bilateral cleft lip surgery (average 8.25, SD 3.41). There was consensus that the model should be made available to all students, residents, specialists and consultants (average 9.57, SD 0.79). The bilateral model was also rated as better than the other models they were familiar with (average 9.50, SD 1.51). Questions about realism in anatomical structures (average 7.75, SD 1.51), perioperative planning and simulation (average 7.57, SD 1.13) and overall realism of the model (average 7.43, SD 1.27) were rated as equally as good as the unilateral model.

### Evaluation of the event

The theoretical component of the program, delivered on the first day, was evaluated positively. Participants rated it as predominantly helpful (average 4.50, SD 0.70) and indicated that the allocated time was largely sufficient (average 3.10, SD 0.32). The second day comprised practical surgical simulations on porcine snouts. The introductory sessions to the unilateral and bilateral models were considered highly beneficial (unilateral: average 4.70, SD 0.48; bilateral: average 4.67, SD 0.50). Overall, the practical training was rated as particularly helpful (average 4.70, SD 0.68), with the duration likewise judged as appropriate (average 3.10, SD 0.32). A detailed overview of the evaluation outcomes is presented in Table 7.

## Online course for students

### Evaluation of the unilateral and bilateral ex vivo cleft lip model

As expected, the students had no practical experience in cleft surgery (unilateral: average 0.20, SD 0.63; bilateral: average 0.00, SD 0.00). General theoretical knowledge of cleft surgery and knowledge of cleft lip closure procedures were at a low level (Table 8). Both models improved general understanding (unilateral: average 8.70, SD 0.82; bilateral: average 8.33, SD 1.58) and the theoretical knowledge of cleft surgery was improved (unilateral: average 7.20, SD 1.36; bilateral: average 6.67, SD 1.32). Not only did the students feel that their theoretical knowledge was deepened (unilateral: average 9.30, SD 1.06; bilateral: average: 8.89, SD 1.83), but also their overall practical skills regarding cleft surgery could be improved (unilateral: average 8.80, SD 1.03; bilateral: average: 8.67, SD 1.73). In addition to the cleft-specific improvements in their practical skills, they found that the models improved their basic surgical skills at least a little (unilateral: average 6.10, SD 2.42; bilateral: average 5.89, SD 2.61). For both models, there is a high level of agreement that the models should be made available to every (dental) student (unilateral: average 9.20, SD 1.62; bilateral: average 8.00, SD 3.43) and that they could imagine using them for course preparation (unilateral: average 9.10, SD 0.99; bilateral: average 9.00, SD 1.58). It seems that there is a considerable demand among students for cleft surgery courses during their curriculum (unilateral: average 8.70, SD 1.64; bilateral: average 8.33, SD 1.94).

### Assessment of the surgical outcome

The performance of the students on the bilateral model showed a higher average of 1.18 SD 0.98 compared the performance of the students on the unilateral model, whose average was 0.91 (SD 0.74).

## Face-to-face course for students

###  Evaluation of the unilateral and bilateral ex vivo cleft lip model

As in the online course, the students´ practical experience in cleft surgery, as well as their general theoretical knowledge and knowledge of the procedure, were at a low level (Table 8). However, the students indicated that they were able to improve their theoretical knowledge and practical skills in the face-to-face course, achieving results that surpassed those of the online course: especially with regard to practical skills as the basic surgical skills (unilateral: average 7.24, SD 2.64; bilateral: average 7.06, SD 2.67), as well as specific skills in cleft surgery (unilateral: average 9.41, SD 1.00; bilateral: average 9.24, SD 1.35). In comparison with the other course, similar or even better results were obtained when the students were asked whether the models should be made available to every (dental) student, whether they would use them for course preparation, or whether they would like to see more courses on cleft surgery in their curriculum.

###  Evaluation of the surgical outcome and tasks

With an average of 1.26 (SD 0.77), the students in the face-to-face group achieved better results in performing cleft lip closure on the unilateral model than the online group. Even better results can be seen when looking at the bilateral model, where there the students achieved an average of 1.62 (SD 0.62).

Table 6 shows the results of the tasks. The students showed an improvement in their performance after the surgical simulation of cleft closure on both models.

**Table 6:** Mean scores achieved by students before and after the surgical procedure in the face-to-face course

Tasks to test basic anatomy of cleft surgery	Points achieved before the exercise	Points achieved after the exercise
First task (naming anatomical points of a unilateral cleft lip), max 7 points	4.44	6.07
Second task (allocation of points of a bilateral cleft lip), max 7 points	2.31	3.44

## Discussion

Because of the lifelong implications for children, cleft surgery should only be performed by experienced surgeons. It is difficult for inexperienced surgeons to grasp the complexities of cleft lip surgery. The purpose of the ex vivo cleft lip models is to help the surgeon gain a basic understanding of cleft lip surgery. They have been found to be suitable models for beginners in oral and maxillofacial surgery to learn cleft surgery [3, 4]. 

In this study, the ex vivo cleft lip models were evaluated by experienced oral and maxillofacial surgeons and dental students. The expertise of the experts allowed a more reliable assessment of the tissue structure of the nasolabial region: The results show that the tissue similarity and anatomical characteristics of the ex vivo cleft lip models are realistic compared to the nasolabial region of children with cleft lip. The main differences with this region are the simulation of the nose, the magnification of the region and a thicker epidermis [3, 4]. Nevertheless, the ex vivo cleft lip models are suitable for simulating the lip closure procedure. This study supports our hypothesis that the models are beneficial for experienced surgeons, residents, and students to learn the basics of lip closure. Experienced and advanced surgeons may benefit in the following ways: In addition to learning modifications to techniques they are already familiar with, they can also try out and train completely new surgical techniques. The models make it possible to estimate how certain incisions, rotations, etc. will affect the lip, philtrum, cleft, and non-cleft sides. This means that step-by-step improvements can be made by testing and refining the technique on the models. Naturally, the ex vivo cleft lip models cannot fully replicate the surgical experience in humans, as the circumstances in the operating theatre differ considerably. Moreover, the pig snout models are flatter than the nasolabial region of cleft lip patients, and they can only reproduce the preparation of cartilage and maxilla to a limited extent. However, they can provide confidence in surgical planning and marking, the order of surgical steps, and the surgical technique itself. This is true for both experienced and inexperienced surgeons. This is supported by the opinion of the experts that the models should be made available to a wide range of people. In one of the questionnaires, a participant stated that before attending our symposium he had not known of any models that simulated cleft lips. We therefore took the opportunity of the professionals’ evaluations to introduce the models to dental students. Firstly, the students support the view of the specialists to make the models available to other students and to include cleft surgery exercises in the curriculum. Students can benefit from the models in many ways: As mentioned in the questionnaires, they felt that the theoretical knowledge was improved by performing cleft closure. The improvement in the students’ task scores on the day of the face-to-face course day serves as a corroborating indicator of this subjective perception. In addition to their use in teaching of cleft surgery, the models can also be used to train basic surgical skills. These features make the models an effective tool for use at all stages of the educational process, whether one is at the novice or specialist level. The low cost of the porcine snout disc allows its inclusion in the dental curriculum despite the often limited financial resources available for education and training. This situation may be even more critical in low-income countries.

In unilateral cleft lip surgery, the non-cleft side is often considered the ideal model for the construction of the cleft side [12]. However, in addition to the cleft side, the non-cleft side is also subject to changes due to surgical modifications: If the philtrum edge is lengthened on the cleft side, the non-cleft side also shortens significantly [12]. Similar results can be seen in our model: Both experienced and inexperienced cleft surgeons lengthened the philtrum on the cleft side and shortened it on the non-cleft side. The data show a tendency for the inexperienced participants to have less change in length on the non-cleft side but no statistical significance was found. At the same time, the experienced surgeons seemed to achieve a slightly smaller difference between the two sides, leading to more harmonious results in practice, but again no statistical significance was found. The small group sizes may be one reason for the lack of significant differences between the results of the groups with higher and lower self-reported skill levels. Another reason may be the subjective nature of the participants’ skill levels. Some participants may have underestimated or overestimated their own skill level compared to others. Furthermore, some participants were highly experienced with numerous operated cases, while others had comparatively limited experience in cleft lip closure. Rogers-Vizena et al. also reported a poor correlation between level of training and surface symmetry in a cohort of residents and fellows with varying levels of experience in craniofacial or paediatric plastic surgery [10]. Nevertheless, they rated high-fidelity cleft surgery models as suitable for evaluating surgeon performance in cleft surgery [10]. With regard to the present study design, an even more accurate analysis could have been obtained by performing a 3D scan of the pig’s snout after marking, as the points may have deviated minimally from the markings on the template due to marking inaccuracies. We decided not to scan after marking and before surgery for the following reasons: First, as we wanted to create a treatment procedure that was as realistic as possible, the time between marking and surgery should not be interrupted by a pause for scanning. Secondly, marking the anatomical points leads to a better understanding of the model and the technique used, so it should not be prepared in advance, but performed by the surgical trainee. Due to the limited surgical experience of the students, a precise three-dimensional analysis of the results was not carried out. Rather, an evaluation of the snouts was made, considering their anatomical correctness and the extent of the student’s understanding of the principle. As previous studies have shown before, especially during COVID-19, online courses for teaching surgical skills can be beneficial for students [13]. In our study, the students’ subjective evaluation of the ex vivo cleft lip models in combination with the courses is at a high level in both courses. However, there seem to be slightly better trends in the evaluation of the face-to-face course groups.

The evaluation of the snouts shows that better results are achieved when students receive personal attention, in contrast to their performance in online courses. The online course was delivered in a synchronous mode via Zoom, which allowed students to interact with the lecturers. A notable disadvantage of Zoom for lecture delivery is the imposition of a pace that participants are expected to follow. This pace is often set by the lecturer and is likely to result in a limited opportunity to comprehend complex topics. Especially when the practical exercises take place immediately after theoretical introduction of the models and the surgical technique used. Step-by-step instructions for the cleft closures were available for both student courses. The combination of synchronous and/or asynchronous teaching and face-to-face course days with the opportunity to interact with the lecturers could be beneficial for the students in the follow-up of the cleft lip closure on the ex vivo models [14]. On the other hand, online courses offer advantages where there are few staff or no space available for such courses, as well as when the participants are located in different places.

As demonstrated in earlier studies, the analysis of 3D-datasets is poised to become the future for evaluating the nasolabial region of children with cleft lips. The major benefit of 3D analysis is its ability to facilitate more precise evaluations in comparison to 2D photographs [11, 15]. As we want to establish the 3D analyzes of children with cleft lip and palate we choose to abstain from any photographic analysis.

The Trios 4 intraoral scanner from 3Shape was used to generate the 3D data. This scanner has a higher accuracy than other scanners designed for the facial region [11]. Another advantage of the Trios 4 scanner is its portability: it can easily be taken to the patient, e.g. in the operating theatre or in the consulting room. It has also been established on ex vivo cleft lip models, making it well suited to our measurement projects [11]. New generations of oral scanners are even more mobile, making them even more flexible to use.

A limitation of our study is the small group sizes together with inhomogeneous groups in the specialist’s course. Due to capacity limits of our skills lab at this time, larger groups were not possible. To form more homogeneous groups, it would be necessary to survey the surgeons’ experience in advance for future studies. This survey could encompass, for instance, the aggregate number of cleft lip operations performed, or the number of cleft lip operations performed per year.

## Conclusion

The evaluation of the specialists and student questionnaires suggests that the models are suitable for use by a wide range of users, from students to residents and specialists. Unlike other high-fidelity models, the ex vivo cleft lip models are inexpensive and easy to obtain. In addition, the models are not only useful for novice cleft surgeons: Experienced surgeons can use the models not only to improve their skills, but also to modify their existing techniques. Combined with the results of our previous studies, we conclude that the tissue characteristics, the ability to perform a variety of surgical techniques and the easy availability of the models make them suitable for training students, residents and cleft surgeons at different levels of training worldwide, including in low-income countries [3, 4]. 

## Data Availability

The data sets used and/or analyzed during the current study are available from the corresponding author upon request.

## Appendix

**Table 7:** Questions and answers of the specialists regarding the unilateral and bilateral ex vivo cleft lip model on a scale of 0-10 ("0=does not apply", "5=partially applies" and "10=completely applies"). The mean of all answered questions (unilateral model n=10, bilateral model n=8) is given as well as the standard deviation

Questions on the ex vivo cleft lip models for specialists	Unilateral model	Bilateral model
	Average (SD)	Average (SD)
I already have general theoretical previous knowledge of cleft surgery	8.30 (2.00)	8.12 (2.85)
I know the procedure for uni-/bilateral cleft lip closure in theory	8.00 (2.49)	8.00 (2.98)
I already have practical experience in uni-/bilateral cleft surgery	5.30 (3.77)	4.00 (5.01)
My general understanding of cleft surgery was improved by practicing on the model	9.50 (0.71)	7.75 (3.33)
My theoretical general knowledge of uni-/bilateral cleft lip surgery was improved by the practice model	7.50 (2.76)	8.00 (3.34)
Only for specialists and senior physicians with prior knowledge in cleft surgery: I was able to deepen/consolidate my previous knowledge through the practical exercise	8.40 (3.06)	8.00 (3.61)
My overall practical skills regarding uni-/bilateral cleft lip surgery were improved by the practice model	8.30 (3.06)	8.25 (3.41)
I consider the model overall a suitable exercise to improve my skills in cleft surgery	8.40 (3.06)	8.14 (3.67)
The model should be made available to every student, resident specialist and senior physician	9.70 (0.66)	9.57 (0.79)
Only for specialists and senior physicians with previous experience with other surgical models for cleft surgery: this model has a clear added value compared to previous models	9.33 (1.16)	9.50 (1.51)
Tissue resemblance: How well could the porcine model simulate the anatomical and tissue properties of the human nasolabial region	7.00 (1.01)	7.57 (1.51)
Realistic planning and surgical procedure: How well could the porcine model simulate preoperative planning and the surgical procedure	7.80 (1.23)	7.57 (1.13)
Overall realism: General realism of the model	7.70 (1.25)	7.43 (1.27)

**Table 8:** Questions and answers of the specialists on the whole event. The mean of all answered questions is given, as well as the standard deviation (SD)

Questions on the event for specialists	Average		SD
Possible answers from “1 = not helpful” to “5 = very helpful”			
The case studies presented were…	4.70		0.48
The presentations were…	4.60		0.52
Overall, the theoretical part was…	4.50		0.71
The presentation on the unilateral cleft model was…	4.70		0.48
The presentation on the bilateral cleft model was…	4.67		0.50
Overall, the practical part was…	4.70		0.68
There were 5 possible answers: “1 = too short”, “2 = short”, “3 = appropriate”, “4 = long”, “5 = too long”			
The temporal scope of the case studies was…		3.20	0.42
The temporal scope of the lectures was…		3.00	0.47
Overall, the temporal scope of the theoretical part was…		3.10	0.32
The time to edit the unilateral cleft model was…		3.10	0.57
The time to edit the bilateral cleft model was…		3.00	0.50
Overall, the temporal scope of the practical part was…		3.10	0.32

**Table 9:** Questions and students' answers about the unilateral and bilateral cleft lip model on a scale of 0-10 ("0=does not apply", "5=partially applies","10=completely applies"). The mean of all answered questions (online course n=11, face-to-face course n=17) is given as well as the standard deviation (SD)

Questions on the ex vivo cleft lip models for students	Unilateral model		Bilateral model	
	Online course	Presence course	Online course	Presence course
	Average (SD)	Average (SD)	Average (SD)	Average (SD)
I already have general theoretical previous knowledge of cleft surgery	2.90 (1.79)	3.71 (2.47)	2.67 (2.12)	3.06 (2.05)
I know the procedure for uni-/bilateral cleft lip closure in theory	2.60 (2.22)	3.06 (2.25)	2.11 (2.15)	2.59 (2.03)
I already have practical experience in uni-/bilateral cleft surgery	0.20 (0.63)	1.29 (2.31)	0.00 (0.00)	1.18 (1.88)
My general understanding of cleft surgery was improved by practicing on the model	8.70 (0.82)	8.71 (1.36)	8.33 (1.58)	8.76 (1.52)
My theoretical general knowledge of uni-/bilateral cleft lip surgery was improved by the practice model	7.20 (1.36)	7.82 (1.67)	6.67 (1.32)	8.06 (1.68)
I was able to deepen/consolidate my previous knowledge through the practical exercise	9.30 (1.06)	8.88 (1.46)	8.89 (1.83)	9.00 (1.37)
My overall practical skills regarding uni-/bilateral cleft lip surgery were improved by the practice model	8.80 (1.03)	9.06 (1.35)	8.67 (1.73)	9.00 (1.46)
I consider the model overall a suitable exercise to improve my skills in cleft surgery	9.00 (1.25)	9.41 (1.00)	9.11 (1.05)	9.24 (1.35)
I was able to improve my basic surgical skills	6.10 (2.42)	7.24 (2.64)	5.89 (2.61)	7.06 (2.67)
I could imagine using the model privately or as direct preparation for surgery courses if required	9.10 (0.99)	9.35 (1.00)	9.00 (1.58)	9.41 (1.23)
The model should be made available to every (dental) student	9.20 (1.62)	9.18 (1.60)	8.00 (3.43)	8.94 (1.81)
I would generally like to see more surgical exercises on cleft surgery in my studies	8.70 (1.64)	9.65 (0.70)	8.33 (1.94)	9.44 (1.03)

## References

[1] Kantar RS, Alfonso AR, Ramly EP, Diaz-Siso JR, Breugem CC, Flores RL. Simulation in cleft surgery. Plast Reconstr Surg Glob Open. 2019. 10.1097/gox.0000000000002438. 31942398 10.1097/GOX.0000000000002438 PMC6908384

[2] Campbell A, Costello BJ, Ruiz RL. Cleft lip and palate surgery: an update of clinical outcomes for primary repair. Oral Maxillofac Surg Clin North Am. 2010. 10.1016/j.coms.2009.11.003. 20159477 10.1016/j.coms.2009.11.003

[3] Lutz R, Kesting MR, Weber M, Olmos M, Tasyürek D, Möst T, et al. An ex vivo model for education and training of bilateral cleft lip surgery. BMC Med Educ. 2023. 10.1186/s12909-023-04575-9. 37596574 10.1186/s12909-023-04575-9 PMC10436624

[4] Lutz R, Schulz KL, Weber M, Olmos M, Möst T, Bürstner J, et al. An ex vivo model for education and training of unilateral cleft lip surgery. BMC Med Educ. 2023. 10.1186/s12909-023-04667-6. 37828467 10.1186/s12909-023-04667-6 PMC10571449

[5] Rogers-Vizena CR, Saldanha FYL, Hosmer AL, Weinstock PH. A new paradigm in cleft lip procedural excellence: creation and preliminary digital validation of a lifelike simulator. Plast Reconstr Surg. 2018. 10.1097/prs.0000000000004924. 30511984 10.1097/PRS.0000000000004924

[6] Taher A, Chow J, Kwon MS, Hunter D, Lucewicz A, Samarawickrama C. Determining the learning curve for a novel microsurgical procedure using histopathology. BMC Med Educ. 2022. 10.1186/s12909-022-03407-6. 35509098 10.1186/s12909-022-03407-6 PMC9066982

[7] Metcalfe J. Learning from errors. Annu Rev Psychol. 2017. 10.1146/annurev-psych-010416-044022. 27648988 10.1146/annurev-psych-010416-044022

[8] Rogers-Vizena CR, Saldanha FYL, Sideridis GD, Allan CK, Livingston KA, Nussbaum L, et al. High-fidelity cleft simulation maintains improvements in performance and confidence: a prospective study. J Surg Educ. 2023. 10.1016/j.jsurg.2023.08.010. 37679288 10.1016/j.jsurg.2023.08.010

[9] Krimmel M, Kluba S, Dietz K, Reinert S. [Assessment of precision and accuracy of digital surface photogrammetry with the DSP 400 system]. Biomed Tech (Berl). 2005. 10.1515/bmt.2005.008. 15832575 10.1515/BMT.2005.008

[10] Rogers-Vizena CR, Yao CA, Sideridis GD, Minahan L, Saldanha FYL, Livingston KA, et al. Cleft lip repair competence can be evaluated with high-fidelity simulation. Plast Reconstr Surg Glob Open. 2022. 10.1097/gox.0000000000004435. 35923989 10.1097/GOX.0000000000004435 PMC9307303

[11] Olmos M, Matta R, Buchbender M, Jaeckel F, Nobis CP, Weber M, et al. 3D assessment of the nasolabial region in cleft models comparing an intraoral and a facial scanner to a validated baseline. Sci Rep. 2023. 10.1038/s41598-023-39352-7. 37500683 10.1038/s41598-023-39352-7 PMC10374634

[12] Massenburg BB, Mercan E, Ettinger RE, Tse RW. The Yin and Yang of primary unilateral cleft lip and nose repair: balance through opposing cleft and noncleft side changes. Plast Reconstr Surg. 2023. 10.1097/prs.0000000000010091. 36541846 10.1097/PRS.0000000000010091

[13] Oetter N, Möst T, Weber M, Buchbender M, Rohde M, Foerster Y, Bauerschmitz C, Röschmann N, Adler W, Rau A, Meyerolbersleben M, Kesting M, Lutz R. COVID-19 pandemic and its impact on dental education: digitalization - progress or regress? Example of an online hands-on course. BMC Med Educ. 2022. 10.1186/s12909-022-03638-7. 35915461 10.1186/s12909-022-03638-7 PMC9340732

[14] Goob J, Erdelt K, Güth JF, Liebermann A. Dental education during the pandemic: cross-sectional evaluation of four different teaching concepts. J Dent Educ. 2021. 10.1002/jdd.12653. 34046898 10.1002/jdd.12653 PMC8242849

[15] Olmos M, Backhaus J, Weber M, Matta R, Vogl C, Schulz K, et al. 3D anthropometry of the nasolabial region in children aged 3 to 9 months as reference database for clinical assessment. Sci Rep. 2025. 10.1038/s41598-025-11024-8. 40721459 10.1038/s41598-025-11024-8 PMC12304160

